# Cultural evolution and US agricultural institutions: a historical case study of Maine’s blueberry industry

**DOI:** 10.1007/s11625-017-0508-3

**Published:** 2017-11-11

**Authors:** Samuel P. Hanes, Timothy M. Waring

**Affiliations:** 1 University of Maine, 5773 South Stevens Hall, Orono, ME 04469 USA; 2 University of Maine, 200 Winslow Hall, Orono, ME 04469 USA

**Keywords:** Cultural evolution, Cultural multilevel selection, Environmental history, Agricultural history, Blueberries

## Abstract

This paper presents a study of the emergence of environmental management institutions in Maine’s blueberry industry. We follow a cultural evolutionary approach to understand the factors that influenced the emergence of these institutions in environmental collective action problems. Specifically, we use a cultural multilevel selection framework to explore the prediction that collective action and institutions of environmental management emerge when cultural selection is the strongest among social groups positioned to solve a given collective action problem. To do this, we construct an evidence typology suited for a historical evolutionary analysis. We find that the scale of cultural adaptation responded to scale of the most pressing adaptive problem. The case study provides support for the group-level selection theory of institutional evolution, and displays patterns of institutional adaptation that respond to changing conditions over time. We argue that the dominant level of selection concept in multilevel selection theory helps to clarify how matches and mismatches between resource scale and institutional scale arise. We conclude that cultural evolutionary theory provides a general causal framework for organizing evidence, and complements the study of environmental history, which provides the temporal depth needed to examine evolutionary hypotheses.

## Introduction

This article explores the historical emergence of environmental institutions in the American agriculture. We are interested here to understand both why and how environmental management institutions emerged. The historical studies of institutional emergence can help us consider possible responses to sustainability challenges today. In approaching this topic, we draw on recent research in the field of cultural evolution, which helps to characterize the historical process of institutional emergence. We also take a case study approach, focusing on Maine’s blueberry industry. New collective agricultural institutions emerged in the wake of major pest outbreaks. These include a state inspection system, an experimental farm, and an industry association. Here we aim to better understand why each institution emerged, when and how it did. For example, why was an experimental farm created in 1944 rather than in 1923?

Cultural evolutionary theory (Henrich 2015) is useful to understand the evolution of institutions generally. We employ a cultural multilevel selection framework (Waring et al. 2015) specifically designed to help explain the emergence of institutions and the spread of behaviours in environmental collective action problems.

Agricultural institutions are critical in determining sustainability outcomes. This is why Cash (2001) explored Cooperative Extension, the main US agency disseminating agricultural science, as a model for the practice of sustainability science. Some of the most important US agricultural institutions are inspection systems, experimental farms, and producer associations. All three are common and crucial for US agriculture, and therefore fundamentally connected to environmental sustainability in the United States. Cultural multilevel selection theory (CMLS) is especially well-suited for understanding how agriculturalists overcome collective challenges, due to the factors that influence cooperation within and among the groups involved. Farmers face numerous environmental and competitive challenges. Both sorts of problems confront the individual farmer, but they also occur at larger social and spatial scales, and require larger groups or even industries to solve it .

In the following section, we outline how the cultural multilevel selection framework for sustainability analysis can be directed towards analysing collective action and institutional emergence. In section “Maine’s blueberry industry”, we use this evolutionary approach to analyse the history of the Maine blueberry industry and the emergence of blueberry agricultural institutions during its two most threatening crises, in 1923 and 1944. Finally, we discuss the challenges and prospects for using an evolutionary framework to study American agricultural institutions generally.

## The cultural multilevel selection framework

The study of collective action (Olson 1965; Ostrom 1990) has become an important component of research on environmental behaviour and resource use. We argue that collective action can often be an outcome of a process of cultural evolution.

The study of cultural evolution is the scientific effort to understand the changes in the variation in human cultural traits (e.g. behaviour, beliefs, norms, language, technology, institutions and traditions) as a result of their influences on the people who exhibit them (Mesoudi et al. 2006). Cultural selection, the cultural equivalent of natural selection, occurs when one cultural trait (such as buying organic coffee) proliferates among some population of people more than an alternative (such as conventional coffee) because of its effects (such as a prestige effect) on those adopting it. For example, some food taboos may have evolved as a cultural protection against dangerous toxins in certain foods (Henrich and Henrich 2010). Cultural evolution is analogous to the process of genetic evolution, but it operates via different mechanisms, such as imitation, and on a shorter time scale (Perreault 2012). Cultural evolution has special relevance for the study of collective action problems, because developing new institutions and norms that support cooperation is one of the main ways that humans solve collective problems. Researchers have proposed that the human capacity for solving group-level adaptive challenges and collective action problems is itself the product of a process of gene-culture coevolution amongst human groups (Boyd and Richerson 2009). Building on the foundation of humans as a uniquely cooperative species, Waring et al. (2015) present a simplified conceptual framework that emphasizes the evolution of cooperative cultural traits within social groups. Their cultural multilevel selection framework is most useful for studying collective action problems and social dilemmas over environmental resource use. A social dilemma is a situation in which the best action for an individual conflicts with the best outcome for the group. Thus, individual cooperation is required to overcome the dilemma and achieve the group-benefits of long-term environmental sustainability at the cost of constrained individual consumption.

Collective action is simply a coordinated action by a group of individuals towards some common goal and may not require individual cooperation. Collective action problems may sometimes be resolved in ways that benefit both groups and individuals directly, whereas social dilemmas, by definition, require that individuals forgo their best option to achieve the best group outcome. Therefore, collective action represents a larger category in which to study the emergence of group behaviour than social dilemmas specifically. Nonetheless, the basic group-level selection processes apply equally to both collective action problems (the general case in which group solutions are easier and more likely) and social dilemmas (the narrow case in which individual freeriding makes group solutions more difficult and less likely).

Group-beneficial behaviours and institutions are thought to emerge when cultural selection operates on the group level, a process referred to as a cultural group selection (Richerson et al. 2016). Although documenting cultural group selection can be complex (see Kline et al., this issue), the process itself is intuitive. In a nutshell, if evolutionary selection pressures are stronger on groups than on individuals, group-beneficial behaviours and institutions are more likely to evolve than individualistic behaviours. Waring et al. (2015) propose the subsidiary hypothesis: “if the strength of selection on groups for resource conservation outweighs the strength of selection on individuals for greater consumption, costly conservation practices and group-beneficial policies can emerge.” In this paper, we collect historical evidence regarding this hypothesis in the history of blueberry agriculture in Maine.

The cultural multilevel selection framework for sustainability analysis is built around a focal cultural trait (e.g. solar panel purchase) or a set of related traits (e.g. energy conservation behaviours). The focal trait must be described within an organizational environment, perhaps with multiple nested levels of social organization, each with some degree of autonomy (e.g. fishermen in towns, in states, and in countries). Focal traits can therefore include institutions, such as a municipal ordinance to lessen pollutants in storm water runoff, or behaviours, such as voluntary adoption of less toxic cleaners and pesticides. To determine the likelihood of cultural group selection, researchers must compare the strength of cultural selection for the focal trait across multiple levels of social organization (see Waring et al. 2015). Finally, historical evidence must be collected to estimate the changes in the selection pressure at each level over time. It is this final step that offers the greatest opportunity for methodological syntheses with the study of environmental history.

Concretely defining relevant groups is a key part of a multilevel evolutionary analysis. The fundamental question is which sort of groups are most significant in the emergence of institutions, and why? Here we consider any groups that try, successfully or otherwise, to create and shape agricultural institutions to combat collective threats. It is the population of such groups (here farmer groups) within which we suppose agricultural institutions evolve.

We might suspect that a rule for restricted resource harvesting to be a group-level cultural adaptation. However, many evolutionary processes can result in individually costly behaviour. So, to confidently identify a group-level cultural adaptation requires that we must determine it as emerged by a process of cultural selection on groups for the specific effects it conveys. In other words, we must identify the process (cultural group selection) to determine if the product is in fact a group-level cultural adaptation. There are two broad categories of evidence for cultural group selection. First, there are the factors that are required for cultural group selection to function at all. These preconditions include: (1) a group-structured cultural trait, (2) group-structured trait outcomes, and (3) mechanisms by which that trait is spread among groups. The second category of evidence is composed of indicators that a given trait is a group-level cultural adaptation. These include (4) the existence of in-group cooperation—costly individual action with clear group benefits, (5) individual behaviours that support cooperation (such as coordination, task specialization, punishment of free-riders), and (6) the strong group culture or supporting institutions. These indicators are suggestive rather than conclusive, but increase the likelihood that the focal trait is a group-level cultural adaptation. When quantitative data are not available, the best available proxies may be used. For example, indicators of the strength of competition between individuals or groups may be a proxy for selective pressure. For a detailed explanation of how to gather empirical evidence for a cultural multilevel selection analysis, see Kline et al. (this issue). In the following sections, we make use of this evidence typology to make historical inferences.

## Maine’s blueberry history

### Background

Today, most cultivated blueberries are “highbush” varieties grown as an orchard crop. Here we discuss “lowbush” blueberries, mostly *Vaccinium angustifolium*, a native, uncultivated plant that grows naturally in acidic, poor soils, and only harvested in Maine and maritime Canada.

Blueberries have a long history—Native Americans burned land to encourage blueberry growth (Munson 1899; Day 1959). The first commercial harvesting was recorded in the 1840s, and canning began around 1870 (Merchant 1929), growing into a significant practice in the 1880s (Woods 1915; Patch and Woods 1922). The industry was then confined to “the barrens,” large areas in eastern Maine that are still the industry’s centre. Before 1920, almost all blueberry canneries were located in or around the barrens (Woods 1915). As early as the 1890s, farmers began converting old farms into blueberry fields, or clearing upland forests for the same purpose, and by the 1920s, mid-coast Maine had significant acreage (Day 1959). The barrens formerly covered more territory, variously estimated from 60,000 to 160,000 hectares by observers at the time (Munson 1899; Woods 1915) compared to approximately 24,000 today (USDA 2014). These were harvested as commons for local consumption until the Civil War stimulated the canning industry. The increased demand for a commercial crop led the private owners to assert land rights in the 1870s (Day 1959). Likely as a result of property rights, there do not appear to have been any significant social dilemmas in the nineteenth century. Today there are approximately 400 growers in Maine; there were more in the past when farms were smaller (Rose et al. 2013). Prior to the 1923 maggot fly outbreak described below, blueberry growers did little to improve their crop besides burning fields to reduce vegetative growth and encourage flowering. In fact, lowbush blueberries remained a non-scientifically improved crop in Maine until the 1920s, even though the Hatch Act had been providing federal funds to found state agricultural experiment stations, since 1887. However, it was not until the 1940s after a crisis that the Maine blueberry industry created an experimental farm. Therefore, a major aim of this paper is to ascertain why an experimental blueberry farm emerged 50 years after the Hatch Act made it possible, and not sooner.

### The 1923 maggot fly crisis

The first major crisis in the industry’s history began after an unusually large infestation of the blueberry maggot fly (*Rhagoletis mendax* Curran) in 1923. The infestation appears to have been a state-wide problem because the governments of other states threatened to use the US Pure Food and Drug Act of 1906 to ban Maine blueberries due to the number of maggots they contained (Smith 1980). As a result, the Maine blueberry industry faced a collective challenge to change the quality or the perception of quality of their exported fruit. In this case, we focus on the proliferation of practices among canners and growers that helped to overcome this state-wide collective action problem.

Cannery owners (i.e. canners) responded rapidly to the maggot fly infestation. Canners met and appointed a committee to make recommendations. The committee conferred with Frank Washburn, Commissioner of the Maine Department of Agriculture, A. Soule, Maine’s Chief of the Division of Inspection, and a US Department of Agriculture food inspector (Day 1959). Canners then adopted two new voluntary practices: a new sorting process to remove infected berries and a new inspection regime in which each canner’s entire product would be inspected for the next 3 years, and labelled as inspected. B.J. Howard of the USDA developed the sorting and cleaning method to help blueberry harvests pass inspection (Lathrop 1939). Canners placed berries in a revolving wire mesh tube and rinsed them with water. Infected berries were softer and were crushed by the water and other berries. Crushed berries fell through the wire. Canners then had their berries inspected and labelled cans as “Packed, inspected, and passed under Maine Pure Food and Drug Law.” The cannery practices reduced fly larvae in canned berries and the inspection and labelling conveyed this to consumers. Both new canning practices spread rapidly. When a second large fly infestation occurred in 1924, a great deal of the harvest was left on the barrens or harvested but never sold. The new sorting process was expensive, slow, and crushed some good berries, yet almost all canners had adopted the practice by 1929 (Day 1959). So, in 1926 Washburn stated, “canners have made lavish expenditures in the perfection of their plants and products” and Chief Inspector Soule said, “it is extremely gratifying to report a marked improvement has been made in the general quality of the product” (Day 1959). Although both Washburn and Soule had some incentive to exaggerate success, the canners’ strategies appear to have worked, as other states never sued Maine and the complaints about poor-quality Maine blueberries ceased.

Grower’s responses to the fly appear to have spread more slowly, possibly because of the need for agronomic science to determine effective techniques to control the infestation over whole growing seasons. Prior studies of the fly had begun in 1915 and University of Maine scientists published a life history study in 1922 (Patch and Woods 1922). Following the 1923 crisis, the committee appointed by the canners requested Warner Morse, Director of the University of Maine’s Agricultural Experiment Station, to study the fly, which resulted in a series of recommendations for the growers. Here we focus on the two main practices they recommended: field burning and insecticide application. Farmers already burned their fields after harvest every 2–4 years at this time. Burning stimulated growth and increased yields, but the plants only produced fruit beginning the second year after a burn. The year immediately after a burn was called the “growth year” because the plants grew but did not produce fruit. Cooperative Extension held a series of meetings in 1923 to emphasize the importance of uniform burning using light straw or hay cover. This eliminated skipped places that produced fruit that flies fed on in the growth year. They also recommended burning all adjacent fields the same year, so flies would have no food in that area in the growth years.

The second practice was the timed application of insecticide. The USDA sent Frank Lathrop, a senior entomologist who became a Maine Extension scientist, to study the problem in 1925 and he began extensive investigations to find effective insecticides (Smith 1980). County Extension agents would monitor and warn growers when fruit flies were emerging from the pupae, but had not yet laid eggs, and thus when “dusting” with pesticides should commence. The committee’s final measure was to ask Maine Cooperative Extension Service to warn growers about the dangers of fly infestation and to promote effective management. In general, these new grower practices spread more slowly than those of the canners. Dusting with pesticides was not widespread until roughly 1940 (Day 1959; Smith 1980), and putting whole fields on a single burning cycle was not widespread until the 1970s (Rose et al. 2013). Together though, these practices were highly successful by 1950 (Lathrop 1952; Drummond and Collins 1999).

These general patterns are suggestive of the decision environment canners and growers faced. Canners could reap all the benefits of sorting, inspecting and labelling their own fruit. Doing so gave them an advantage over non-adopting canners, who would lose sales to the labelling adopters. Thus, canners were individually motivated to adopt the new practices independently and immediately.

Growers also benefitted from controlling the pest on their fields; in doing so they likely had to consider their neighbours’ actions. Both burning and dusting eliminated the fruit flies in one’s field, but if one’s neighbours failed to do either, then fruit flies would recolonize quickly. So, there was no benefit to early adoption. However, there were costs for being late because late adopting growers could cause reinfestation on the neighbours’ fields and suffer social recrimination. The slowest spreading technique, burning neighbouring fields the same year, required one farmer to make a sacrifice for another. One would have to burn a year later or early and this would reduce production for them. Therefore, growers did benefit individually from these practices, but multiple factors likely led them to synchronize activities with neighbours.

In summary, there was a collective response to the drop in sales due to the fly infestation. So, although growers needed to coordinate, it appears that individual canners and growers both eventually gained by adopting the new best practices, thereby creating additional beneficial effect on the perception of Maine’s blueberries in general. In the 1923 case, there was a collective action problem for state labelling and inspection, and quite possibly a coordination game among growers to control the pest, but no social dilemma for either growers or canners.

### The 1944 armyworm crisis

Between the outbreaks of the 1920s and 1944, there was a failed attempt to expand blueberry institutions in 1932. Unfortunately, few documents related to this episode remain. The USDA set up a small laboratory in Cherryfield, Maine in the late 1920s to study the maggot fly. It closed in 1932. When it did, at least two prominent canners (who were also large-scale growers) asked the University of Maine to take it over. The Director of the Experiment Station and the University President favoured doing so, but the University’s Board of Trustees voted against it citing budget constraints. The canners then approached the State Legislature without success. The State government was heavily in debt due to the great depression, and legislators were looking for ways to raise revenue and cut expenses. The depression was also hurting the industry because consumers had reduced blueberry purchases. Prices had fallen by three-quarters and most canneries had curtailed production and laid-off workers (Deering 2017). Thus, in the 1930s and 1940s, state-level organizations were not willing or able to fund scientific research on blueberry production, even during an industry crisis.

The 1944 blueberry crisis in Maine probably stemmed from the confluence of increased market competition and a pest outbreak. Competition in the domestic fruit industry had influenced blueberry production for decades. For instance, New Jersey and Michigan had begun planting highbush blueberries in the 1920s (Dow 1950), and blueberry production in those states was now mature. By the 1940s, canners and growers considered highbush blueberry crops in other states to be a major source of competition (Day 1959). The Maine industry even blamed out of state competition for the low prices from 1942 to 1944 (Day 1954). On top of new competition, poor weather conditions created localized crop losses in 1942 and 1943. Then, in 1944, blueberry yield fell from 17 million bushels the previous year to 3.5 million (Yarborough 2014) largely due to an armyworm (*Mythimna unipuncta* Haworth) outbreak. In this case study, we focus on the evolution of political support for three things: a new experimental farm, a self-imposed tax to fund crop science on it, and a committee to represent the collective interests of the industry.

A subset of packers (processors were now called “packers”) contacted Arthur Deering, Dean of Agriculture at the University of Maine. He then led the effort to persuade the rest of industry to work together. Agricultural editor Clarence Day stated that, “That was no easy task” (Day 1959). Deering organized a meeting on December 5, 1944 in Ellsworth to address the crisis. Although growers did not tend to see one another as competitors, there was a split between processors. Several cooperatives formed in the late 1920s and these “created intense feelings and competition” with the packing companies. Deering invited leaders of each company and cooperative. And, although public, Deering did not announce the meeting and only invited a limited group (about 40 persons) to increase the chance of reaching a consensus (Day 1954). It is clear that the industry had strong internal competition between companies and cooperatives, and overcoming this required careful balancing of representation.

At the meeting, industry members asked questions about the armyworm, but made it clear that their main need was for more science. Day (1954) paraphrased some of their concerns from the meeting’s minutes:

Does the armyworm work more on new burns or on second year bushes? Have you raised a plant from seed? Does mulch prevent winter injury? Is the domestic bee a good pollinator? Does freezing destroy insects? What is the effect of continuous application of insecticide on bee populations? What is the experience regarding heavy burning?

These questions illustrate the hope with which industry members sought scientific solutions for a wide range of agricultural challenges they confronted.

Deering also invited two key State Senators. Senator Dunbar discussed his experience obtaining support for Maine’s Aroostook Experimental Farm and proposed introducing similar legislation for a blueberry farm. He also suggested a blueberry tax to pay for the research on it. Industry members agreed that they used “illogical methods” and badly needed long-term research and saw Aroostook Farm as valuable to potato growers. Many felt, though, that research was an essential need, and whether a farm was the best way to do this or not, they did not know. In the end, however, a large majority supported an experimental farm and tax (Day 1954).

The State Legislature quickly passed two laws at the industry’s behest: one to purchase an experimental farm and the other to establish Maine’s “blueberry tax” to fund research at the farm. The industry advocated for the tax; it was essentially a self-tax. Both bills passed easily (Eastport Sentinel 1945a, b). The laws required the University President to appoint committees to locate the farm and advise the University of the industry’s research needs. The committee purchased what would become the Blueberry Hill Farm Experiment Station, and the research advisory committee has since expanded and is now known as the Wild Blueberry Commission of Maine.

The bill to create the blueberry farm offers additional evidence of direct transmission of solutions between industries. The State Legislature had previously purchased two experimental farms in Maine: Highmoor Farm, founded in 1907 with an emphasis on apple experiments, and Aroostook Farm, founded in 1913 for potato and wheat growers in Northern Maine. The State used Hatch Act funds to buy Highmoor, but used its own tax dollars to buy Aroostook. Senator Dunbar was in the State Legislature in 1913 and was one of the leaders in gathering support for Aroostook. The blueberry farm bill drew directly from the text of the Aroostook bill. They had the same section organization, both beginning with “To this end, there shall be purchased, stocked and equipped for the use and benefit of… a suitable experimental farm, situated as the committee on selection and purchase hereinafter named shall determine,” and the two bills even carried the same names: An Act to Provide for Scientific Investigation a.) in Agriculture in Aroostook County, b.) with Blueberries. This copying is direct evidence of the transmission of a group-level cultural trait (the act and farm) between agricultural groups in the larger population of US farmer groups and agricultural industries. Evidence of cultural transmission of this quality is exceedingly rare.

### Evolutionary analysis of institutional emergence in Maine’s blueberry history

If the industry’s responses to the blueberry crises of 1923 and 1944 represent group-level cultural adaptations, we should observe historical evidence of group-level evolutionary factors leading to their emergence. Collective action, defined as a group-beneficial and coordinated action, clearly occurred among industry members in both epochs. By applying the empirical rubric, we seek to clarify whether the collective action observed was due to group-level cultural selection or individual level pressures.

Our review of the historical evidence suggests that a social dilemma, real or perceived, existed in 1944 but not in 1923. Although we do not have precise quantitative information on costs and benefits, the state-wide meetings and a campaign to rally support for the farm suggests that a social dilemma may have indeed existed, otherwise no rallying would have been necessary. Other factors may also have contributed to the perception of a dire social dilemma and the willingness to cooperate. The armyworm was the second major outbreak in recent times, and many saw armyworm damage as part of a recurring state-wide challenge of insect pests caused by lack of scientific research. Also, unlike in 1923, there was no quick technical fix that could alleviate group pressure shared by the entire industry. The 1923 case had also shown the industry how valuable crop and pest science could be.

To apply the rubric, we first evaluated preconditions for cultural group selection and then indicators of group-level cultural adaptation (see Table 1). When applying the rubric to the 1923 case, all preconditions for cultural group selection appear to be at a local, individual social scale. Individual canners and growers adopted the focal traits (sorting/inspection/labelling, burning/dusting); these were all at the level of the individual business. The social scale of these traits outcomes’ was also individual or local. Although we have no direct evidence of imitation, this seems to have taken place at the individual business level as well.

**Table 1:** Evidence for selection on focal practices across levels of social organization

	Metrics	1920s	1940s
(A) Preconditions for cultural group selection			
Group-structured cultural trait	Social scale of trait variation	Business	State
Group-structured outcomes	Social scale of trait-based outcomes	Local (fly) individual (canners)	State
Group-structured trait spread	Scale of imitation, competition	Businesses	industries (tax/farm)
(B) Indicators of group level cultural adaptation			
Cooperation and collective action	Social scale of collective problem	Local (fly) state (sales)	State/industry
	Cooperative trait (indiv. cost)	✓	✓ (tax)
	Cooperative trait (group benefit)	X	✓
Supporting behaviours	Individual coordination	✓	✓
	Resource-specific usage rules	✓	✓
	Punishment of non-cooperators		
	Monitoring of resource use		✓
	Social markers		
Supporting institutions	User group boundaries	Prior	Prior
	Resource boundaries	Prior	Prior
	Self-governance methods	Prior	Prior
Decision environment		Individual (canners) coordination (growers)	Dilemma (industry)
Selection on	Businesses	**++**	−
	State/industry	+	**++**
Dominant level of selection		Businesses	Industry

Evidence for cultural selection can be either evidence that the preconditions for cultural group selection are met (A) or evidence that cultural group selection has occurred, resulting in group-level cultural adaptations (B). Evidence for group selection is denoted with a check mark, evidence against with X. The direction of selection at each level is denoted as positive (+) or negative (−)

Indicators of group-level cultural adaptations are also mixed or negative in 1923. The new canner practices carried short-term individual costs in terms of the loss of harvested fruit, and canners did not require much coordination or cooperation with each other. Not all canners immediately adopted sorting, inspection and labelling, which shows that canners who did sell their own berries regardless of what other canners did. Canners that adopted sorting, inspecting and labelling early could reap individual benefits. Moreover, these solutions appeared to display efficiencies of scale. For example, a larger subgroup of canners would have been more likely to be heeded by politicians, and more able to assemble the resources to start research on improved methods. So, although the fruit fly infestation effected the entire industry, there was no sign of a social dilemma—improvements in product quality benefitted individual canners directly and the industry generally. Furthermore, the voluntary nature of adoption is evidence there was no social dilemma; institutions to sanction or punish were not needed. It appears that there was no strong social dilemma between canners, individual and group interests aligned and new practices were both collectively and individually beneficial, and spread via individual level mechanisms. For growers, a similar set of circumstances unfolded. Burning and dusting appear to have had mostly individual benefits. Growers took longer to fully adopt dusting and burning, and in the latter case this may have been because of the additional coordination required between growers to put neighbouring fields on the same burning cycle. Canners’ action also took some pressure off the growers. While some indicators of group-level adaptation are present in 1923, such as individually-costly action, most of the benefits were individual rather than group. Overall, we take this evidence to indicate that in the 1923 crisis the dominant level of selection for agricultural practices for both canners and growers was the individual level.

Applying the rubric in 1944, we see different results. The social scale of the focal traits (tax/farm/association) was at the state/industry scale. Outcomes from the farm would benefit the entire industry as well. The scale of imitation was clearly between industries (i.e. potato to blueberry). The institutions that emerged in 1944 meet the preconditions for cultural group selection and also bear many indications of being group-level adaptations, as the empirical rubric reveals (see Table 1). First, the legislative actions, the experimental farm, the industry tax for crop science and the industry standing committee are all examples of group-level cultural traits, they have no individual equivalent. Second, it appears that individual and group costs and benefits were different than in 1923. For example, fruit flies only harm the berries, whereas the armyworm ate leaves and it took the plants a year or two to fully recover. This raises the cost of inaction by lowering yield the next year as well. The industry also saw the problem as much broader in 1944. They felt they needed more science to prevent disasters like in 1944, but also to increase production more generally. They saw other crops doing this and felt they lagged behind in terms of scientific research. Thus, the real or perceived costs were higher, which could have spurred greater willingness to collaborate.

One strong signal of group-level adaptation is direct individual contributions to collective institutions, such as a tax. The new Maine blueberry tax carried a direct, immediate cost to individuals, and the benefits accrued to the group over the long-term. It is a quintessential example of a cooperative trait, which is unlikely to have emerged via selection on the level of individual businesses. Third, the state-level institutions (tax and farm) were imitated from the Maine potato industry, revealing a pattern of descent with modification, the hallmark of adaptive evolution. Maine potato and apple farmers already had their own versions of these institutions and experimental farms were common across the US by 1944 (Rasmussen 1989). Evidence shows industry members discussed these models explicitly. This is significant for multiple reasons. Primarily, it indicates that the group-level institutional trait of a tax-funded experimental farm to support an agricultural industry was a trait that could be imitated at the time. Perhaps more importantly, it suggests the bounds of the population of industries that could be imitated. Starting with the Hatch Act in 1887, many crops had adopted research farms across the US, and Maine blueberries were quite late in comparison to other industries and states. In Maine, there were two other model systems (potato and apple) available for imitation or other forms of social learning. This is useful because of what we can infer about the nature of the traits those industries adopted, specifically, traits that were likely to help their industry. Thus, the source population is itself evidence of group-level adaptation. Finally, the two bills for Aroostook and Blueberry Hill provide the strongest direct evidence of cultural inheritance, as they show descent with modification.

## Discussion: collective action, evolution and institution building

We assert that collective action and institutions of environmental management emerge when cultural selection for environmental management is strongest among social groups positioned to solve the collective action problem at hand. Of course, our single case study does not allow for a test of this assertion, but it does appear to support the general assertion in part, because the case allows for a comparison between 1923 and 1944. Moreover, this case shows how the CMLS framework might be applied to other situations.

Defining groups appropriately is a central part of the CMLS framework, and difficult to perfect. For example, “farmer groups” are composed of more than just farmers. Farmers, university scientists and government agency officials historically have sought alliances with one another (Hamilton 1990), alliances that often shift and change. While this adds complexity, these complications do not undermine the framework, but help specify how it can be applied in these industries. Another complicating factor is group fluidity. They not only shift over time, but they may simultaneously hold multiple, possibly divergent, purposes. For instance, US highbush and lowbush associations jointly support grants that study environmental problems that affect both crops, such as fungal pathogens. Lowbush growers cooperate with their prime competition in this domain because the gains are potentially quite large. Thus, despite fierce internal competition, cooperation between highbush and lowbush growers gives them a better chance to compete with other fruit crops. The push and pull of cooperation and competition at different levels is precisely where the CMLS framework is most useful.

**Fig. 1:**
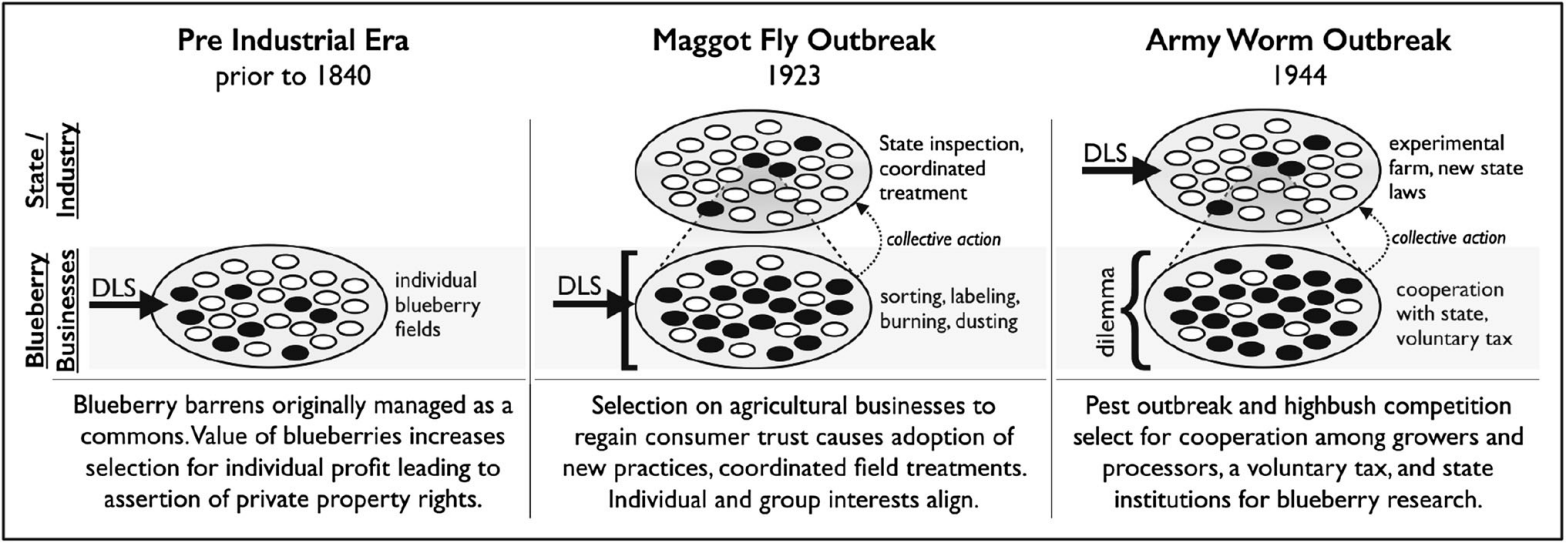
Likely changes in the level of selection for blueberry agricultural practice and policy (ca. 1800–1950). DLS dominant level of selection (hypothesized). White and black ovals represent social units with cooperative and non-cooperative cultural trials, respectively. Square bracket represents coordination problems. Flower braces represent social dilemmas

Perhaps the greatest benefit of an evolutionary approach is in facilitating comparison and generalization across cases. In the case study, we found evidence of group-level cultural adaptation creating new institutions to solve a collective action problem in 1923 and a social dilemma in 1944. But we also observed different institutional and behavioural adaptations emerge in response to different types of selection pressures caused by the changes in the collective challenge itself over time. For instance, canners and growers could address the fruit fly crisis of 1923 without the need for individually-costly cooperation. However, the next pest outbreak was more dire, and required cooperation and institution building in addition to collective action. This was because, in 1944, selective pressure was even greater on the group (see Fig. 1). Indeed, the entire Maine lowbush blueberry industry faced an existential threat. The case reveals how the collective action and collective management institutions have responded over US agricultural history to the social scale of the most pressing challenges that farmers face. We suggest this is a useful lens for understanding the emergence of environmental management institutions generally.

Like other analyses of this sort, we have assumed that cooperative solutions emerge by cooperation spreading over time among individuals and groups. However, humans can often foresee collective adaptive challenges and avoid the costs of cooperation through planning and communication. In this way, an entire group may transition to a new institutional solution at once. Thus, a group-level crisis might simply trigger an adaptive group-level response in individuals. Although Waring et al. (2015) acknowledged that humans are pre-adapted in this manner, it is worth noting that such adaptive responses are a different mechanism, and may operate faster than the basic group selection process we apply here.

One unexpected finding was the way the scale of social groups and problems interacted with the scale of resources and legal boundaries. In the 1944 case, the scale of ecological challenges and legislative boundaries coincided conveniently. Most of the lowbush blueberry industry was in Maine and the problem appeared solvable via in-state action. Research on environmental management institutions has highlighted the importance of a match between the scale of an environmental resource and the institutions which manage it (Cumming et al. 2006). For sustainable outcomes, management institutions must operate at a scale on which the resource regenerates and can be preserved. An evolutionary analysis helps by revealing the processes by which scale matches (or mismatches) emerge by identifying the scale of cultural adaptation.

By focusing on cultural adaptation and the strength of selection, we can better see why environmental management institutions evolved the way they did. Our study shows how well-adapted institutions may emerge at the dominant level of selection. For example, the fruit fly outbreak in 1923 caused collective action, but not institution building because the dominant level of selection was at the corporate, not the industry level. This changed in 1944, when the dominant level of selection aligned with the pre-existing state institutions, and the physical scale of lowbush blueberry production. The scale match between institution and resource only occurred when the dominant level of selection coincided with that scale, so that effective institutions evolved.

## Conclusion

This evolutionary framework helps us understand the historical emergence of environmental management institutions in a few specific ways. First, the framework helps to determine the type of social grouping most relevant to the emergence of the focal trait. Second, the framework helps identify the period of time during which the trait emerged, based on the presence of conditions for group-level adaptation. Third, the framework helps in identifying the natural resource boundaries and legal jurisdictions most influential in each case. We acknowledge that these benefits could also result from a thorough non-evolutionary historical analysis. However, the greatest benefit of the CMLS framework is that it helps to systematize the process of historical investigation, making different cases easier to compare. An evolutionary framework also provides a fresh perspective, making phenomena such as group-level cooperation, which many take for granted, worthy of investigation and explanation in their own right. We believe this perspective can shed new light on why and how US farmers and their allies cooperated to find important environmental management institutions.

Our methods led to several insights about data gaps likely in historical research using cultural evolutionary theory. One was that reconstructing historical patterns of imitation was challenging. We reconstructed a plausible sequence of imitation in 1944, from Maine potatoes to blueberries. Future historical use of evolutionary frameworks will likely need to use and refine such proxies. The historical record does not always contain data needed to understand costs and benefits of adoption, and exact pressures and motivations. Resistance to the blueberry tax is a good example. In addition, such gaps will always be greater when studying institutions created by common people, as opposed to elites, because they left fewer documents. Despite these challenges, we feel future historical studies can build robust explanations by applying the CMLS framework in broader comparisons of institutional emergence across multiple crops.
